# Chemical Composition of an Aphid Antifeedant Extract from an Endophytic Fungus, *Trichoderma* sp. EFI671

**DOI:** 10.3390/microorganisms8030420

**Published:** 2020-03-17

**Authors:** Nutan Kaushik, Carmen E. Díaz, Hemraj Chhipa, L. Fernando Julio, M. Fe Andrés, Azucena González-Coloma

**Affiliations:** 1 The Energy Resources Institute, India Habitat Center, Lodhi Road, New Delhi 110003 India; kaushikn2008@gmail.com (N.K.); hrchhipa8@gmail.com (H.C.); 2 Amity University Uttar Pradesh, Sector 125, Noida 201313, India; 3 Instituto de Productos Naturales y Agrobiología, CSIC. Avda. Astrofísico F. Sánchez, 3, 38206 Tenerife, Spain; celisa@ipna.csic.es; 4 College of Horticulture and Forestry, Jhalawar, Agriculture University Kota-, Rajasthan 326001, India; 5 Instituto de Ciencias Agrarias, CSIC, Serrano, 115-dpdo, 28006 Madrid, Spain; lfjuliot@gmail.com (L.F.J.); mafay@ica.csic.es (M.F.A.)

**Keywords:** endophyte, *Trichoderma*, triglyceride, sterol, fatty acid, *Myzus persicae*, culture media, antifeedant

## Abstract

Botanical and fungal biopesticides, including endophytes, are in high demand given the current restrictive legislations on the use of chemical pesticides. As part of an ongoing search for new biopesticides, a series of fungal endophytes have been isolated from selected medicinal plants including *Lauraceae* species. In the current study, an extract from the endophytic fungus *Trichoderma* sp. EFI 671, isolated from the stem parts of the medicinal plant *Laurus* sp., was screened for bioactivity against plant pathogens (*Fusarium graminearum*, *Rhizoctonia solani*, *Sclerotinia sclerotiorum* and *Botrytis cinerea*), insect pests (*Spodoptera littoralis*, *Myzus persicae*, *Rhopalosiphum padi*) and plant parasites (*Meloidogyne javanica*), with positive results against *M. persicae*. The chemical study of the neutral fraction of the active hexane extract resulted in the isolation of a triglyceride mixture (m**1**), eburicol (**2**), β-sitostenone (**3**), ergosterol (**4**) and ergosterol peroxide (**5**). The free fatty acids present in the acid fraction of the extract and in m1 (oleic, linoleic, palmitic and stearic) showed strong dose-dependent antifeedant effects against *M. persicae*. Liquid (potato dextrose broth, PDB and Sabouraud Broth, SDB) and solid (corn, sorghum, pearl millet and rice) growth media were tested in order to optimize the yield and bioactivity of the fungal extracts. Pearl millet and corn gave the highest extract yields. All the extracts from these solid media had strong effects against *M. persicae*, with sorghum being the most active. Corn media increased the methyl linoleate content of the extract, pearl millet media increased the oleic acid and sorghum media increased the oleic and linoleic acids compared to rice. The antifeedant effects of these extracts correlated with their content in methyl linoleate and linoleic acid. The phytotoxic effects of these extracts against ryegrass, *Lolium perenne*, and lettuce, *Lactuca sativa*, varied with culture media, with sorghum being non- toxic.

## 1. Introduction

Endophytes include all microbes present in asymptomatic plant tissues. Among them, the study of fungal endophytes has led to the discovery of a large quantity of bioactive natural products in recent years [1,2,3,4,5,6,7]. Furthermore, some fungal endophytes produce compounds found in their host plants such as azadirachtin, podophyllotoxin, hypericin and taxol [8,9,10,11,12,13]. Therefore, medicinal plants and their endophytes are good candidates for the isolation of bioactive metabolites.

Botanical and fungal biopesticides, including endophytes, are in high demand given current restrictive legislations on the use of chemical pesticides. In this context, endophytes isolated from species of the *Lauraceae* family produced extracts with crop protection effects [14]. For example, *Guignardia mangiferae*, *Glomerella acutata* and *Diaporthe* sp. isolated from *Laurus novocanariensis*, *Persea indica* and *Ocotea foetens*, showed potent insect antifeedant effects against *Spodoptera littoralis*, *Leptinotarsa decemlineata* and *Rhopalosiphum padi*. Furthermore, *G. mangiferae* from *P. indica* showed insect antifeedant effects and strong in vitro nematicidal activity against the root-knot nematode *Meloidogyne javanica*, with the dioxalanone guignardianone D being the nematicial agent with antifeedant effects on insect pests [14,15].

As part of an ongoing search for new biopesticides, a series of fungal endophytes have been isolated from selected medicinal plants including *Lauraceae* species. Here, we report on the isolation of the endophytic fungal strain EFI671 from *Laurus* sp., the chemical characterization of its organic extract and the identification of the bioactive compounds against *Myzus persicae*. Additionally, liquid (Potato Dextrose Broth, and Sabouraud Dextrose Broth) and solid (corn, sorghum, pearl millet and rice) growth media were tested in order to optimize the yield and bioactivity of the fungal extracts.

## 2. Materials and Methods 

### 2.1. Plant Material

The plant material for endophyte isolation was collected from medicinal plant *Laurus* sp., which was purchased from the herbal garden nursery of the University of Agricultural Sciences, Bangalore, India. The samples were placed into sterile polybags and transported, under refrigeration, in a box container until isolation processing within 48 h of collection.

### 2.2. Isolation of Endophytic Fungus Trichoderma sp. EFI 671

Endophytic fungus was isolated from the medicinal plant of *Laurus* sp. according to Kumar et al. (2013) with the surface sterilization method [16]. The surface of stem and leaf was sterilized with 70% ethanol for 2 min followed by 1% sodium hypochlorite for 3 min. Sterilized stem and leaf were dried on a sterile blotting sheet and then chopped on a sterile Petri plate and transferred to Potato Dextrose Agar plates (PDA). These plates were incubated at 24 °C for 3–15 days in a BOD incubator. Growing fungal colonies were transferred to fresh PDA plates to get a pure culture. *Trichoderma* sp. was initially identified by microscopic examination and later identified by molecular methods as described in the next section. The culture was maintained on PDA slant by routine sub culturing. The culture has been deposited in the National Centre for Microbial Resources (NCMR), India.

### 2.3. Molecular Characterization of Trichoderma sp. EFI 671

The genomic DNA of pure fungal isolate *Trichoderma* sp. EFI 671 was extracted using DNeasy Plant mini kit (Qiagen GmbH, Hilden Cat. No 69104) by following the manufacturer’s instructions. The extracted DNA was used for polymerase chain reaction (PCR) amplification by primers ITS1 and ITS4 according to Kumar et al. (2011) [17]. The polymerase chain reaction was achieved in 25 µL of reaction mixture, which contained 2.5 µL of 10× PCR Buffer with 15 mM MgCl_2_ (Applied Biosystem, India), 0.5 µL of dNTP mix (10 mM, Applied Biosystem), 2.5 µL of ITS1, ITS4 primers (10 Pico mole/µL), 1 µL of DNA template, and 0.5 µL of Ampli Teg Gold (5 U/µL). The ITS1 and ITS4 primers were synthesized from Merck nucleotide synthesis services (Bengaluru, India) according to the ITS1 5′TCCGTAGGTGAACCTGCGG3′ and ITS4 5′TCCTCCGCTTATTGATATGC3′ sequence. The PCR was performed on a Veriti Thermal Cycler (Applied Biosystem, Foster City, CA, USA) using the following programs: initial denaturation at 94 °C for 2 min; 30 cycles of denaturation, annealing, and elongation at 94 °C for 1 min, 57 °C for 90 sec, and 72 °C for 2 min followed by final elongation at 72 °C for 4 min. The negative control was also run using sterile water. The amplified product was checked on 1.5 per cent agarose gel-by-gel electrophoresis. The amplified product was sequenced by Merck sequencing services, Bengaluru, India. The identification of strain was done by sequence similarity of amplified sequence with NCBI database using Basic Local Alignment Search Tool (nBLAST). The sequence was aligned and trimmed by DNA Baser 4.2 software based on quality read values. The sequence was submitted to NCBI database. The *Trichoderma* sp. EFI 671 was cultivated on 200 g of sterilized rice medium in a 1000 mL conical flask for metabolite extraction as per the method of Kumar and Kaushik (2013) [18]. After 15 days of growth at 25 °C under dark conditions, metabolites were extracted using 250 mL of ethyl acetate (EtOAc) solvent according Kumar et al., (2013) [16]. The organic solvent was evaporated to dryness in a rotary evaporator (Heidolph, Germany), yielding 500 mg of ethyl acetate extract (EtOAc). Further, EtOAc (350 mg) extract was partitioned with n-hexane (Hex) and 90% MeOH to separate polar and non-polar compounds. The weight of hexane and methanol extract was 180 mg and 150 mg, respectively.

### 2.4. Large-Scale Cultivation for Compound Isolation 

The fungal strain *Trichoderma* sp. EFI 671 was incubated on 250 g sterilized rice medium in 10 conical flasks of 1000 mL volume for 15 days under the same conditions described above for the isolation of active compounds. The extraction and partition of fungal extract was done according to Kumar et al. (2013) as described above [16]. A total of 6.0 g of hexane extract was obtained from the partition of 13.46 g of EtOAc extract.

### 2.5. Optimization of Media

The culture conditions were optimized in liquid and solid media. To determine the best liquid media for active component production, Potato Dextrose Broth (PDB) and Sabouraud Broth (SDB) media were used. The fungal strain was inoculated at small scale in 30 mL of medium and incubated for 7 days. Different solid substrates (corn, sorghum, barley, pearl millet and rice) were also used to determine their effect on the production of active compounds by the endophytic fungi. In each 1 L flask, 200 g of solid substrates were incubated for 20 days as per the procedure of Kumar and Kaushik (2013) [18]. The bioactive compounds were extracted with EtOAc using the same protocol. The fungal growth was measured as biomass estimation and spore count.

### 2.6. Isolation and Identification of Bioactive Compounds

Column chromatography (CC) was performed on silica gel 40–70 µm (Merck), and precoated silica gel 60 F254 (Merck) was used for preparative Thin Layer Chromatography (TLC). Compounds were visualized on TLC by heating after spraying with Oleum reagent (H_2_O:acetic acid:sulfuric acid 16:80:4). Preparative HPLC was performed on a Varian ProStar with a 20 mm × 250 mm Interstil silica column (10 µm particle size). The bioactive hexane extract was dissolved in dichloromethane (DCM), and the solution was neutralized with 0.5N NaOH. The aqueous layer was acidified with 2N HCl at pH 2 and extracted with DCM (3 × 200 mL). Both organic layers were dried with anhydrous Na_2_SO_4_, filtered and concentrated in a vacuum to afford 3.72 g and 110 mg of neutral and acid extracts, respectively. The neutral extract was fractionated by vacuum liquid column chromatography (VLC) over silica gel using hexane/ethyl acetate mixtures of increasing polarity (100:0–50:50 Hex:EtOAc), to afford 11 fractions. Further preparative high-performance liquid chromatography (HPLC) of fraction 1 (208 mg), eluted with an isocratic mixture of Hex:EtOAc (95:5) at 3mL/min, gave mixture **1** (28 mg). Fraction 3 (132 mg) was chromatographed by prep HPLC with an isocratic mixture of Hex:EtOAc (97:3) at 3 mL/min to give mixture **1** (450 mg), compound **2** (5.2 mg) and compound **3** (6.2 mg). Fraction 7 (101 mg) was chromatographed by prep HPLC with Hex:EtOAc (95:5) at 3 mL/min to give compound **4** (15.9 mg). Fraction 11 (39.4 mg) was further subjected to prep-TLC and eluted with Hex:EtOAc (95:5) to give compound **5** (5.7 mg).

The compounds were subjected to NMR spectroscopy and mass spectrometry for structure elucidation. Optical rotations were measured with a Perkin-Elmer model 343 polarimeter. NMR experiments were recorded on a Bruker Advance-400 and AMX2 500 MHz spectrometers. Chemical shifts were calculated using the solvent as internal standard (CDCl_3_, at δ_H_ 7.26 and δ_C_ 77.0). HRESIMS (positive-ion mode) data were obtained using a Micromass LCT Premier and HREIMS on a Micromass Autospec instrument at 70 eV.

In order to identify the acid esters present in compound **1**, 450 mg of fraction 1 was added to 50 mL of a 20 mg/mL K_2_CO_3_ solution in MeOH stirred and monitored by TLC. After 18 h, the reaction mixture was extracted with EtOAc and dried over Na_2_SO_4_ to give an extract (430 mg) that was further chromatographed on a flash column filled with 25 g Si-gel and eluted at 18 mL/min with 1.5%–15% Hex-EtOAc to afford fractions 1A (95.3 mg) and 1M (273.4 mg) that were further analyzed by GC-MS.

### 2.7. Gas Chromatography-Mass Spectrometry (GC-MS) Analysis

Analyses were carried out using a Shimadzu GC-2010 gas chromatograph coupled to a Shimadzu GCMS-QP2010 Ultra mass detector (electron ionization, 70 eV) and equipped with a 30 m × 0.25 mm i.d. capillary column (0.25 µm film thickness) Teknokroma TRB-5 (95%) Dimethyl-(5%)-diphenylpolysiloxane. Working conditions were as follows: split ratio, 20:1; injector temperature, 300 °C; temperature of the transfer line connected to the mass spectrometer, 250 °C; initial column temperature, 70 °C, then heated to 290 °C at 6 °C min^−1^. Electron ionization mass spectra and retention data were used to assess the identity of compounds by comparing them with those of standards or found in the Wiley 229 mass spectral database. Further, the retention times of authentic compounds (isolated in this work and purchased from Sigma Aldrich) were also used to confirm the identities of the constituents. The relative amounts of individual components were calculated based on the GC peak area (FID response) without using a correction factor.

### 2.8. Antifungal Bioassay

The antifungal activity of fungal metabolites against different phyto-pathogens such as *Fusarium graminearum*, *Rhizoctonia solani*, *Sclerotinia sclerotiorum* and *Botrytis cinerea* was procured from the Indian Type Culture Collection (ITCC), IARI, New Delhi. The activity was determined by food poison assay according to Chowdhary and Kaushik (2015) [19]. Dried crude extract and partitioned fungal extract were dissolved in their respective solvent to prepare a stock solution of 40 mg/mL concentration. From the stock solution, 1.00–0.01 mg/mL concentrations were prepared in 10 mL of PDA medium by adding 250 µL, 125 µL, 62.5 µL, 25 µL and 2.5 µL of stock. Intoxicated media plates were inoculated with eight cork borer plugs of plant pathogenic fungi measuring 0.5 cm^2^ placed in the plate. The solvent control was prepared using the respective solvent in 10 mL of PDA media. The plates were incubated at 25 ± 2 °C for 7 days. The growth inhibition (GI) of the phyto-pathogen was recorded after 48 h to 7days or before the overgrowth of control plates. % GI was calculated as:

(1) $$GI\%=100\times \frac{\left(Diameter\;of\;fungi\;in\;control\;plate\;\left(A\right)-Diameter\;of\;fungi\;in\;intoxicated\;plate\;\left(B\right)\right)}{Diameter\;of\;fungi\;in\;control\;plate\;\left(A\right)}$$

### 2.9. Insect Bioassay

Insect colonies maintained at Instituto de Ciencias Agrarias, CSIC, were used to conduct the bioassays. *Spodoptera littoralis*, *Myzus persicae* and *Rhopalosiphum padi* colonies were reared on an artificial diet, bell pepper (*Capsicum annuum*) and barley (*Hordeum vulgare*) plants, respectively, and maintained at 22 ± 1 °C and >70% relative humidity, with a photoperiod of 16:8 h (L:D) in a growth chamber [20].

The upper surface of *C. anuum* and *H. vulgare* leaf disks or fragments (1.0 cm^2^) were treated with 10 μL of the test substance. The crude extracts and products were tested at an initial dose of 10 or 5 mg/mL (100 or 50 µg/cm^2^) respectively. Five Petri dishes (9 cm diam.) or twenty ventilated plastic boxes (2 × 2 cm) with 2 newly molted *S. littoralis* L6 larvae (<24 h) or 10 apterous aphid adults (24–48 h old) each were allowed to feed at room temperature for *S. littoralis* (<2 h) or in a growth chamber for the aphids (24 h, environmental conditions as above). Each experiment was repeated 2–3 times (SE < 10%) and terminated when the consumption of the control disks reached 65%–75% for *S. littoralis* or after 24 h for aphids. The leaf disks were digitalized to calculate the area consumed (Image J, http://imagej.nih.gov/ij). Aphid settling was measured by counting the number of aphids on each leaf fragment. Feeding or settling inhibition (%FI or %SI) was calculated as

(2) % FI/SI=[1−(T/C)×100]

where T and C are the consumption/settling of treated and control leaf disks, respectively. The antifeedant effects (% FI/SI) were analyzed for significance by the nonparametric Wilcoxon signed-rank test. Extracts and compounds with an FI/SI >75% were further tested in a dose-response experiment (34 serial dilutions) to calculate their relative potency (EC_50_, the effective dose to give a 50% feeding/settling reduction) from linear regression analysis (% FI/SI on log-dose) [21].

### 2.10. Nematode Bioassay

A laboratory (ICA-CSIC) root-knot nematode (*Meloidogyne javanica*) population maintained on tomato (*Lycopersicon esculentum* var. Marmande) plants in pots at 25 ± 1 °C and >70% relative humidity was used for the experiments. Second-stage juveniles (J2) hatched within 24 h (from egg masses handpicked from infected tomato roots) were used. The experiments were carried out in 96-well microplates (Becton, Dickinson) as described [22]. The organic extracts and pure compounds were tested at initial concentrations of 1.0 and 0.5 mg/mL, respectively (final concentration in the well), and diluted serially (4–5 concentrations) when needed. The number of dead juveniles was recorded after 72 h. All treatments were replicated four times. The percentage mortality data shown in the tables were corrected according to Scheider-Orelli’s (1947) formula [22].

### 2.11. Phytotoxicity Tests

The experiments were conducted with *Lactuca sativa* and *Lolium perenne* seeds (100 seeds / test) in 12-well microplates as described [21]. The extracts were tested at a concentration of 0.4 mg/mL (final concentration in the well). Germination was monitored for 6 (*L. sativa*) or 7 days (*L. perenne*) and the root/leaf lengths measured at the end of the experiment (25 plants were selected randomly for each experiment, digitalized and measured with the application Image J, http//rsb.info.nih.gov./ij/). An analysis of variance (ANOVA) was performed on root/leaf length data.

## 3. Results

### 3.1. Identification of Endophytic Fungi

The endophytic fungi *Trichoderma* sp. EFI 671 was isolated from the stem parts of the medicinal plant *Laurus* sp. The axenic culture was identified by the rDNA sequencing of the internal transcribed region (ITR) as *Trichoderma* sp. EFI 671, based on the sequence similarity with the NCBI database. The culture showed 99% sequence similarity with *Trichoderma* sp. (Accession no. KY363353.1).

### 3.2. Bioactivity Screening of Endophytic Fungi

Bioactivity screening of *Trichoderma* sp. EFI671 extracts was carried out against the phyto-pathogenic fungi *F. graminearum*, *R. solani*, *S. sclerotiorum*, *B. cinerea*, the nematode *M. javanica* and the insects *S. littoralis*, *M. persicae* and *R. padi*. The results of the bioactivity of the extracts against phytopathogens and pest insects are given in Table 1 and Table 2. The total EtOAc fungal extract and the MeOH partition showed moderate (65% and 50% inhibition, respectively) effects on *S. sclerotiorum* mycelial growth, suggesting the presence of polar antifungal compounds.

The EtOAc extract showed significant settling inhibition effects on *M. persicae* (82% SI) and *R. padi* (73% SI). Its partition into non-polar (Hex) and polar (MeOH) extracts resulted in 87% and 78% SI against *M. persicae* and 39% and 54% SI against *R. padi*, indicating that the hexane extract concentrated most of the antifeedant compounds against *M. persicae*, while the partition decreased the extract effect against *R. padi* (Table 2). Therefore, a larger-scale hexane extract was prepared for bioassay-guided fractionation and compound identification. Both extracts were inactive against root-knot nematode *M. javanica.*

### 3.3. Chemical Characterization of the Extract

The acid extraction of the large-scale hexane extract (Hex) gave two aphid antifeedant fractions, neutral (HexN) and acid (HexA), with the acid fraction being more effective (Table 2). The GC-MS analysis of the hexane extract and its partitions showed that the extract contained linoleic as the main component followed by oleic, palmitic and stearic acids. Methyl linoleate and methyl oleate were present as minor components. The HexA fraction contained the same acids but not their methyl esters (Table 3).

The chemical study of the neutral fraction (HexN) resulted in the isolation of mixture **1** as the major component. The spectroscopic data for mixture 1 were consistent with the structure of a triglyceride (see Supplementary files). The fatty acid composition of the acid (1A) and methylated (1M) fractions obtained from the hydrolysis of mixture **1** was confirmed by GCMS and by the comparison of retention times of methyl esters of authentic fatty acids. The GC-MS analysis showed the presence of oleic, linoleic and palmitic acids as the main components, with stearic acid as a minor one (Table 3). On the basis of the spectral data analysis, mixture 1 contained 1-oleoyl-2-linoleoyl-3-palmitoylglycerol as the major component [23,24].

**Figure 1:**
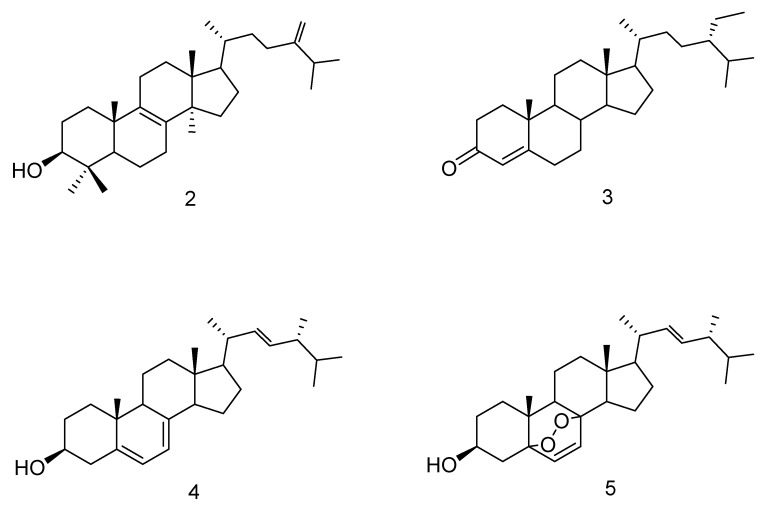
Molecular structures of compounds **2** (4,4,14α,24-tetramethyl-5α-cholesta-8, 24(24′)-dien-3β-ol (eburicol), **3** stigmast-4-ene-3-one (β-sitostenone), **4** (ergosta-5, 7, 22-triene-3β-ol, ergosterol) and **5** ((3β,5α,8α,22E)-5,8-epidioxyergosta-6,22-dien-3-ol, ergosterol peroxide).

Four known sterol compounds were isolated from *Trichoderma* sp. (Figure 1). EFI 671 neutral Hex extract. Their structures were determined based on their ^1^H, ^13^C NMR and MS spectroscopic data (see Supplementary files). Their spectroscopic data were in agreement with those reported for eburicol (4,4,14α,24-tetramethyl-5α-cholesta-8, 24(24′)-dien-3β-ol (**2**) [25], stigmast-4-ene-3-one (**3**) [26], ergosterol (ergosta-5, 7, 22-triene-3β-ol) (**4**) [25] and ergosterol peroxide (3β,5α,8α,22E)-5,8-epidioxyergosta-6,22-dien-3-ol) (**5**) [27].

The components (mixture **1**, compounds **2**–**5**) of the neutral fraction (HexN) were not active against *M. persicae*, while the free fatty acids (1A) and their methylated derivatives (1M) showed strong dose-dependent aphid antifeedant effects (Table 4).

### 3.4. Optimization of Media

Different liquid and solid growth media were tested on the isolate EFI 671 in order to optimize the bioactivity of the extracts against aphids. The liquid media were the conventional Potato Dextrose Broth (PDB) and Sabouraud Broth (SDB). The solid media included corn, sorghum, pearl millet and rice. Table 5 shows the results of the spore count and extract yields. The solid media pearl millet and sorghum gave the highest spore counts, while pearl millet and corn gave the highest extract yields (mg/g).

All the EtOAc extracts from these solid media showed low–moderate settling inhibition effects against *R. padi* and strong effects against *M. persicae*, ranked as follows: sorghum > corn > barley > rice > pearl millet. The conventional liquid media PDB and SDB gave inactive extracts (Table 6).

The different media resulted in quantitative and qualitative differences in chemical composition (Table 7), thus explaining the differences in their bioactivity. Overall, the major components were methyl linoleate and linoleic acid, followed by palmitic and oleic acid. Methylated derivatives of palmitic and oleic acids were only present as minor components in corn and pearl millet. Corn media increased the methyl linoleate content of the extract, pearl millet media increased the oleic acid and sorghum media increased oleic and linoleic acids compared to rice. A multiple correlation analysis between the content of fatty acids and bioactivity against *M. persicae* showed a strong significant correlation with methyl linoleate followed by linoleic acid (Table 7).

### 3.5. Phytotoxicity of Bioactive Extracts

**Figure 2:**
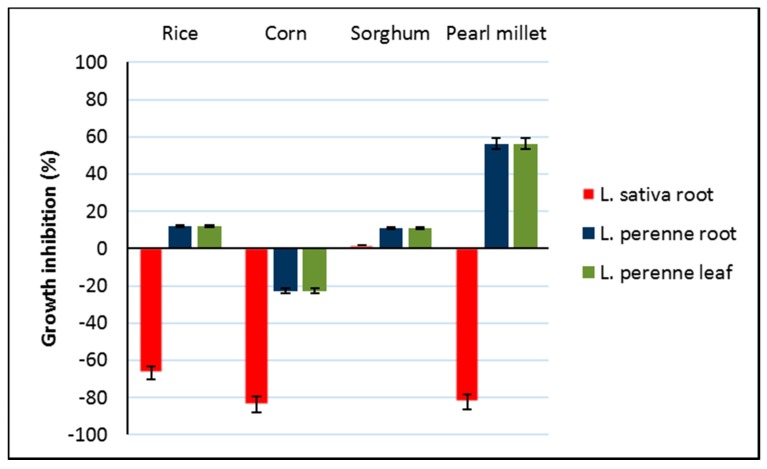
Phytotoxic effects (% inhibition) of the different EFI671 solid media extracts on *Lactuca sativa* and *Lolium perenne*) root and leaf growth. Bars represent the average relative values ± standard error (*n* = 25 plants measured).

The phytotoxic effects of the bioactive extracts were tested on *L. perenne* and *L. sativa* germination and growth. None of these extracts affected the germination of *L. sativa* or *L. perenne* (% inhibition <10). All extracts (rice, corn and pearl millet) inhibited the root growth of *L. sativa* (66%–83%) except for sorghum. Pearl millet extract significantly increased the growth of *L. perenne* root and leaf (60%) (Figure 2). Therefore, the phytotoxic effects of the bioactive extracts could be modulated by the culture media.

## 4. Discussion

Species of the genus *Trichoderma* have been widely used as biocontrol agents because of their myco-parasitic capacity and ability to improve plant protection against phytopathogens [28], including *Trichoderma* endophytes [29]. *Trichoderma* sp. is also an insect biocontrol agent [30,31,32], including endophytic *Trichoderma* [33,34].

The production of effector molecules and secondary metabolites by *Trichoderma* spp. contributes to their beneficial biological activities [35], making this genus a valuable source of a wide variety of secondary metabolites [36]. In this work, a triglyceride mixture with 1-oleoyl-2-linoleoyl-3-palmitoylglycerol [23,24] being the main component (mixture **1**), and four known sterols—eburicol (**2**) [25], β-sitostenone (**3**) [26], ergosterol (**4**) [25] and ergosterol peroxide (**5**) [27]—have been isolated from the neutral hexane extract of the *Trichoderma* endophyte EFI671.

The most important chemical classes reported in *Trichoderma* are anthraquinones, peptaibols, polyketides, pyrones, terpenes and diketopiperazine-like secondary metabolites [36,37,38]. Sterol compounds such as ergosterol, lanosterol and pyrocalciferol were detected for the first time in the fermentation of a *T. pseudokoningii* strain [37]. Extracts of *Trichoderma* sp. isolated from saline lands contained ergosterol and ergosterol peroxide along with other sterol compounds and two new sorbicillin acid analogs [39]. Ergosterol, stigmasterol and β-sitosterol have been isolated from two strains of *T. harzianum* [40]. Recently, ergosterol, cerevisterol and a tryglyceride derivative have been identified from *Trichoderma* sp. Jing-8 strain isolated from the stem of *Panax notoginseng* [41]. However, this is the first report on the isolation of compounds **2** and **3** from *Thrichoderma* sp.

The bioactive compounds from the *Trichoderma* isolate EFI 679 were fatty acids and their methylated derivatives, present in the Hexane extract in free form and as part of the triglyceride **1**, with strong dose-dependent aphid antifeedant effects. Long-chain primary alcohols from *Trichoderma citrinoviride*, such as 1-hexadecanol, affected the feeding preference of the aphid *R. padi* [42], but this is the first report on the production of insect antifeedant fatty acids by a *Trichoderma* endophyte.

Fatty acids are major components of plant lipids and can affect growth and development of insect herbivores and influence host suitability to invasive insects [43]. Additionally, the insecticidal and antifeedant action of fatty acids and esters has been described. Linoleic and oleic acid were insecticidal against fourth instar *Aedes aegyptii* larvae and antifeedant to neonate larvae of *Helicoverpa zea*, *Lymantria dispar*, *Orgyia leucostigma*, and *Malacosoma disstria*, while triglycerides such as 1, 3-dilinoleoyl-2-olein, 1, 3-dioleoyl-2-linolein, and 1, 2, 3-trilinolein were not active [44]. Stearic and palmitic acids have been isolated from *Brassicaceae* bio-oil as antifeedants to Colorado potato beetle [45]. Linolenic and linoleic acids have been reported as antifeedants against *M. persicae* [46]. Furthermore, a mixture of methylated fatty acids was antifeedant to Colorado potato beetle, while the individual components (methyl hexadecanoate, ethyl hexadecanoate, methyl octadecanoate, and methyl icosanoate) were inactive, suggesting a synergistic effect. Hexadecanoate methyl (methyl palmitate) and ethyl (ethyl palmitate) esters were antifeedant against the aphids *M. persicae* and *Diuraphis noxia* [47]. Pentacosyl heptacosanoate has been isolated from the natural wax of the plant *Dolichandra cynanchoides* as an insecticidal agent against *S. frugiperda* and *Epilachna paenulata* [48]. Therefore, fatty acids, which are non-toxic substances, are a promising class of molecules for the control of phytophagous insects. The insect antifeedant mode of action of fatty acids is unknown. However, linoleic acid preferentially bound the *Bemisia tabaci* chemosensory protein (CSP1) in competitive binding assays [49]. CSP proteins (Mp10) are produced by the saliva of the green peach aphid *M. persicae* [50] and therefore could play a role in fatty acid–*M. persicae* interaction. However, further research is needed to support this hypothesis.

Different liquid and solid growth media were tested to optimize the bioactivity against aphids of the fungal extracts. Medium optimization is a useful method to enhance the yield of pharmaceutical metabolites in endophytes. Precursor/adsorbent feeding is a strategy to enhance the desired metabolite yield [51]. For example, tryptamine increased campothecin yield and cellulose (paper disk) increased mycoepoxydiene [52]. The different solid media resulted in quantitative and qualitative differences in fatty acid and ester composition and had strong settling inhibition effects against *M. persicae* that correlated with methyl linoleate and linoleic acid. Therefore, differences in amino acid composition, carbohydrate and cellulose content between the different solid media used could be responsible for the differences in fatty acid yields.

There are no reports on the phytotoxic effects of fatty acids. The media-dependent selective phytotoxic effects of the EFI671 extracts could be related to the presence of sterols. For example, ergosterol peroxide (compound **5** produced by EFI 671) has strong allelopathic effects reported against the grass *Echinochloa crus-galli* [53].

## 5. Conclusions

An endophytic fungi *Trichoderma* sp. EFI 671 was isolated from the stem parts of the medicinal plant *Laurus* sp. The chemical study of its bioactive EtOAc extract resulted in the isolation of a triglyceride mixture **1**, eburicol (**2**), (24R)-stigmast-4-ene-3-one or β-sitostenone (**3**), ergosterol (**4**) and ergosterol peroxide (**5**). Free fatty acids present in the Hex extract and mixture **1** (oleic, linoleic, palmitic and stearic) showed strong dose-dependent aphid antifeedant effects against *M. persicae*. Different liquid (PDB and SDB) and solid (corn, sorghum, pearl millet and rice) growth media were tested in order to optimize the yield and bioactivity of the fungal extracts. Pearl millet and corn media gave the highest extract yields. All the EtOAc extracts from these solid media had strong effects against *M. persicae*, with sorghum being the most active. Corn media increased the methyl linoleate content of the extract, pearl millet media increased the oleic acid and sorghum media increased the oleic and linoleic acids compared to rice. Their antifeedant effects correlated with methyl linoleate and linoleic acid. The phytotoxic effects of these extracts varied with culture media, with sorghum being the least toxic. Thus, fatty acids can be used as antifeedants for aphid control. However, formulated *Trichoderma* extracts should be tested in vivo followed by scaled-up production and optimization processes.

## Figures and Tables

**Table 1:** Screening of bioactivity of *Trichoderma* sp. EFI 671 ethyl acetate (EtOAc), methanol (MeOH) and hexane (Hex) extracts against plant pathogens and parasites at 1 mg·mL^−1^ concentration. Data are expressed as % mycelium growth inhibition (fungal pathogens) and % mortality (*M. javanica*).

Extract	*Fusarium graminearum*	*Rhizoctonia solani*	*Sclerotinia sclerotiorum*	*Botrytis cinerea*	*Meloidogyne javanica*
	% Inhibition				% Mortality
**EtOAc**	**03.95 ± 8.46**	**43.40 ± 05.33**	65.4 ± 03.50	09.8 ± 05.26	6.21 ± 1.29
MeOH	10.12 ± 8.95	45.30 ± 05.38	50.5 ± 11.38	22.2 ± 12.90	2.73 ± 0.60
Hex	08.56 ± 6.10	28.05 ± 18.04	05.6 ± 15.50	30.8 ± 06.53	7.50 ± 1.21

**Table 2:** Insect antifeedant effects of *Trichoderma* sp. EFI 671 extracts (EtOAc), solvent partitions of the EtOAc extract (MeOH, Hex) and acid partition of the Hex extract (HexN, HexA) ^$^ against insects.

Extract	*Myzus persicae*	*Rhopalosiphum padi*	*Spodoptera littoralis*
	%SI ^a^ (100 µg/cm^2^)		%FI ^a^ (100 µg/cm^2^)
EtOAc	82.52 ± 6.19 *	72.63 ± 5.99 *	17.29 ± 07.80
MeOH	76.89 ± 7.97 *	54.25 ± 6.71	11.66 ± 07.30
Hex	87.42 ± 5.32 *	39.79 ± 7.80	30.01 ± 15.94
HexN	76.63 ± 7.30 *		
HexA	97.10 ± 1.30 *		

^$^ HexN is the neutral hexane extract fraction and HexA is the acidic hexane extract fraction. ^a^ Percentage settling (%SI, *n* = 100 insects)/feeding (%FI) inhibition (*n* = 20 insects); * Significantly different from the control (*p* < 0.05), Wilcoxon paired rank test.

**Table 3:** GC-MS analysis of the EFI671 hexane extract (Hex), its acid fraction (HexA), the free fatty acids mixture (1A from the hydrolysis of 1) and their methylated esters (1M).

Retention Time (min)	Compound	Hex	HexA	1A	1M
		(% Abundance)			
23.57	Hexadecanoic acid methyl ester	0.78	-	-	19.72
	(methyl palmitate)				
24.19	Hexadecanoic acid	15.89	26.70	24.70	-
	(palmitic acid)				
26.41	Octadecadienoic acid methyl ester	2.89	-	-	36.35
	(methyl linoleate)				
26.50	Octadecenoic acid methyl ester	0.67	-	-	39.25
	(methyl oleate)				
26.90	Octadecanoic acid methyl ester	-	-	-	4.68
	(methyl stearate)				
27.03	Octadecadienoic acid	42.60	43.05	20.76	-
	(linoleic acid)				
27.12	Octadecenoic acid	32.30	27.47	46.15	-
	(oleic acid)				
27.45	Octadecanoic acid	3.90	2.78	8.39	-
	(stearic acid)				

- Could not be detected.

**Table 4:** Antifeedant effects of mixture **1,** the free fatty acids from the hydrolysis of mixture **1** (1A), their methylated esters (1M) and compounds **2**–**5** on *Myzus persicae*.

Extract/Compound	*Myzus persicae* %SI ^a^ (50 µg/cm^2^)	EC_50_^b^ (µg/cm^2^)
**1**	35.86 ± 8.4	
1A	85.56 ± 5.3 *	6.87 (4.34–23.8)
1M	72.67 ± 6.9 *	1.03 (0.18–5.64)
**2**	15.35 ± 6.94	
**3**	18.33 ± 7.09	
**4**	17.08 ± 6.7	
**5**	38.76 ± 7.5	

^a^ Percent settling (%SI, *n* = 100 insects) inhibition, ^b^ Concentration needed to produce 50% setting inhibition (EC_50_) against *M. persicae* and 95% confidence limits (lower-upper), * Significantly different from the control (*p* < 0.05), Wilcoxon paired rank test.

**Table 5:** Spore count and extract yield from the different culture media (liquid and solid) of EFI 671. PDB: Potato Dextrose Broth; SDB: Sabouraud Broth.

Media	Extract Yield (g/mL)/(g/g)	Spore Count/mL
PDB	00.02	5.04 × 10^6^
SDB	00.07	8.96 × 10^6^
Sorghum	09.84	7.92 × 10^8^
Barley	03.56	3.95 × 10^8^
Corn	13.16	4.27 × 10^8^
Pearl millet	22.64	9.29 × 10^7^
Rice	06.48	2.88 × 10^8^

**Table 6:** Bioactivity of EFI 671 extracts from different media against *Myzus persicae* and *Rhopalosiphum padi*.

Extract	*Myzus persicae* %SI ^a^ (100 µg/cm^2^)	EC_50_^b^ (µg/cm^2^)	*Rhopalosiphum padi* %SI ^a^ (100 µg/cm^2^)
Rice	87.16 ± 3.22 *	33.54 (26.12–42.30)	64.49 ± 5.58
Corn	88.6 ± 5.22 *	14.63 (08.98–23.82)	30.43 ± 7.23
Sorghum	90.19 ± 2.28 *	1.39 (0.01–2.00)	62.62 ± 5.76
Barley	85.38 ± 5.32 *	23.10 (14.07–37.58	54.59 ± 7.53
Pearl millet	70.31 ± 5.45 *	38.57 (26.46–54.36)	40.62 ± 8.75
PDB	41.08 ± 8.71	~100 ^c^	64.17 ± 6.78
SDB	51.08 ± 7.64	~100 ^c^	na

^a^ Percent setting (%SI, *n* = 100 insects) inhibition, ^b^ Concentration needed to produce 50% settling inhibition (EC_50_) against *M. persicae* and 95% confidence limits (lower-upper). ^c^ Estimated values. * Significantly different from the control (*p* < 0.05), Wilcoxon paired rank test, na = data not available due to low amount of extract.

**Table 7:** Gas chromatography–mass spectroscopy (GC-MS) analysis of the different EFI 671 solid and liquid media extracts and correlation coefficients (CC, p = significance level) with bioactivity (EC_50_ in µg/cm^2^ for *M. persicae*).

Growth Medium		% Abundance					M. persicae EC_50_ (µg/cm^2^)
	Methyl Palmitate	Palmitic Acid	Methyl Linoleate	Linoleic Acid	Oleic Acid	Methyl Oleate	
Rice	-	25.87	30.12	28.19	3.01	-	33.54
Corn	0.84	14.23	41.67	29.45	3.85	2.12	14.63
Sorghum	-	11.34	34.19	36.28	5.50	-	1.39
Barley	-	21.50	29.49	23.30	3.09	-	23.10
Pearl millet	1.13	17.77	26.89	35.16	11.74	2.29	38.57
PDB	-	4.47	0.86	-	-	-	100.0
SDB	-	4.49	0.96	0.83	0.18	-	100.0
CC (p)	−0.272 (0.555)	−0.599 (0.155)	−0.969 * (0.0003)	−0.932 * (0.002)	−0.497 (0.255)	−0.298 (0.516)	

* Significantly different from the control (*p* < 0.05), Wilcoxon paired rank test. - Not detected.

## Supplementary Materials

Supplementary materials can be found at https://www.mdpi.com/2076-2607/8/3/420/s1. A file containing the spectra and spectroscopic data of the isolated compounds and pictures of the relevant antifeedant bioassay has been provided.
