# Supplementary material for: Chemical Composition of an Aphid Antifeedant Extract from an Endophytic Fungus, Trichoderma sp. EFI671
Source: Microorganisms. 2020 Mar 17;8(3):420. doi: 10.3390/microorganisms8030420 (PMC7143094; doi:10.3390/microorganisms8030420)
Supplement: Supplementary file 1 [file microorganisms-08-00420-s001.pdf]

Supplementary data

Chemical Composition of an and Aphid Antifeedant Extract from an Endophytic Fungus *Trichoderma* sp. EFI671

Nutan Kaushik [1,2], Carmen E. Díaz [3], Hemraj Chhipa[1,4], Luis F. Julio[5], M. Fe Andrés[5] and Azucena González-Coloma[5,*]

[1] *The Energy Resources Institute, Indian Habitat Center, Lodhi Road, New Delhi, India;*

[2] *Amity University, Uttar Pradesh, Sector 125, Noida, India;*

[3] *Instituto de Productos Naturales y Agrobiología, CSIC. Avda. Astrofísico F. Sánchez, 3, 38206. Tenerife, Spain;*

[4] *College of Horticulture and Forestry, Jhalawar, Agriculture University Kota-326001, Rajasthan, India;*

[5] *Instituto de Ciencias Agrarias, CSIC, Serrano, 115, 28006 Madrid, Spain;*

* *Correspondence: azu@ica.csic.es*

Contents

Figure S1. ^{1}H and ^{13}C NMR spectra of mixture **1**

Figure S2. ^{1}H and ^{13}C NMR spectra of compound **2**

Figure S3. HREIMS spectrum of compound **2**

Figure S4. ^{1}H and ^{13}C NMR spectra of compound **3**

Figure S5. HREIMS spectrum of compound **3**

Figure S6. ^{1}H and ^{13}C NMR spectra of compound **4**

Figure S7. HREIMS spectrum of compound **4**

Figure S8. ^{1}H and ^{13}C NMR spectra of compound **5**

Figure S9. HREIMS spectrum of compound **5**

Table 1. ^{13}C NMR data of compounds **2-5**

Figure S10. Antifeedant bioassay against *Myzus persicae*

Figure S11. Phytotoxicity bioassay against *Lolium perenne* and *Lactuca sativa*

Mixture **1:**

^{1}H NMR (500 MHz, $CDCl_3$): δ 0.88 (9H, m), 1.27 (56H, m), 1.60 (8H, m), 2.02 (8H, m), 2.31 (6H, td, *J*=7.6, 3.3 Hz), 2.77 (2H, t, *J*=6.7 Hz), 4.14 (2H, dd, *J*=11.9, 6.0 Hz), 4.29 (2H, dd, *J*=11.9, 4.3 Hz), 5.26 (1H, tt, *J*=6.0, 4.3 Hz), 5.35 (6H, m); ^{13}C NMR (100 MHz): δ 14.1, 22.7 – 34.2 , 62.1, 68.9, 127.9, 128.1, 129.7, 130.0, 130.0, 130.2, 172.8, 173.2, 173.3; HRESI-TOFMS: *m/z* 879.7407 $[M+Na]^{+}$, calcd for $C_{55}H_{100}O_6Na$

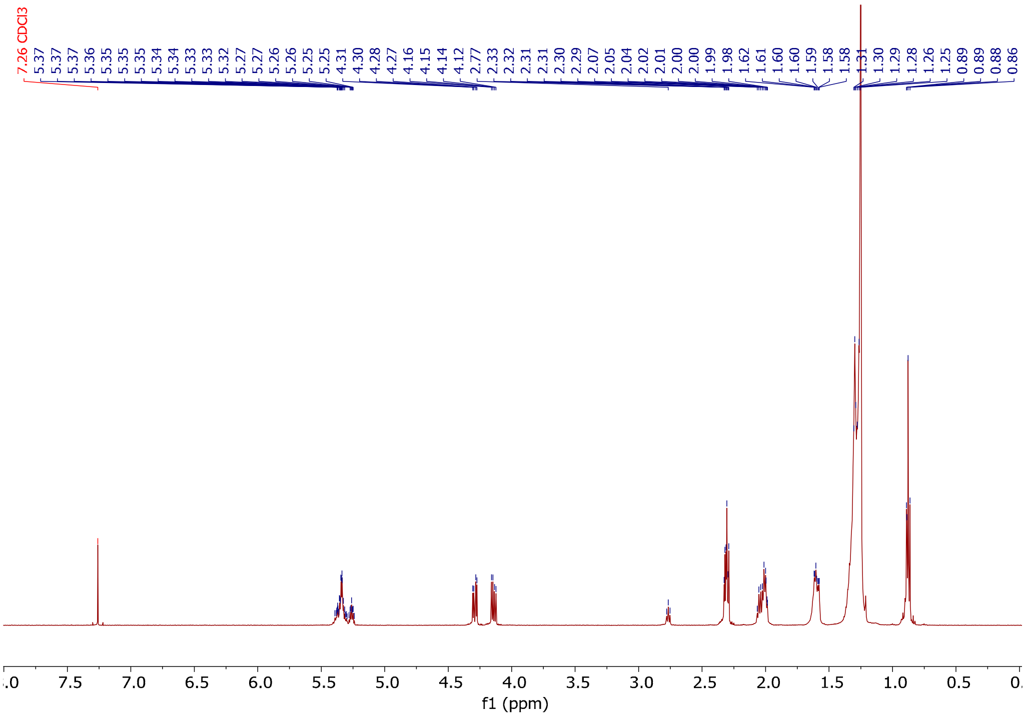

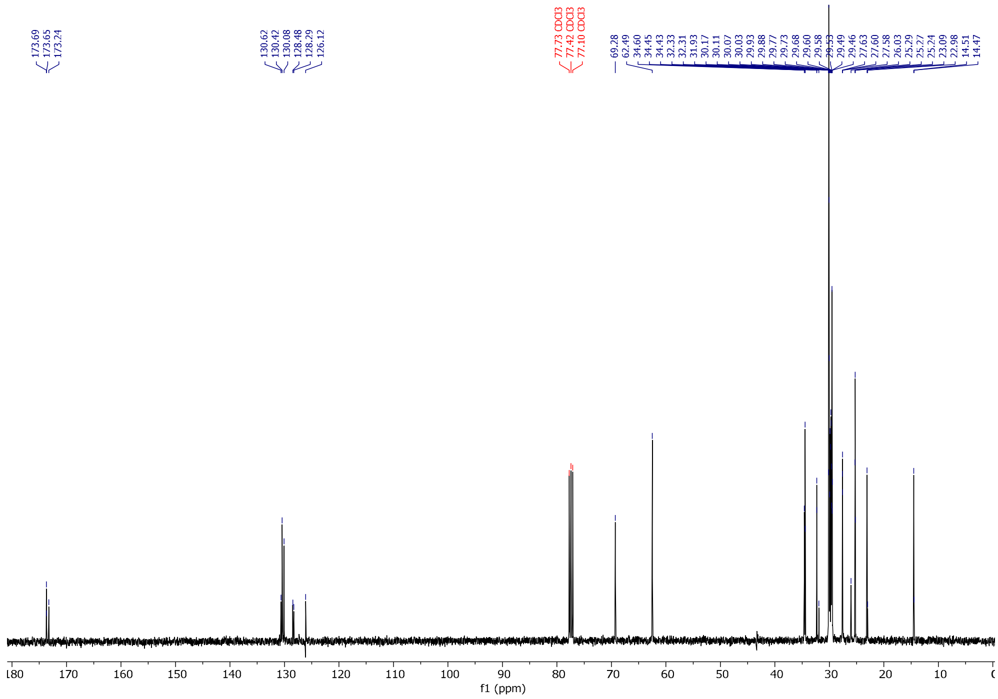

Figure S1. ^{1}H and ^{13}C NMR spectra of compound **1**

Compound **2:**

^{1}H NMR (500 MHz, $CDCl_3$): δ 0.69 (3H, s, H-18), 0.81 (3H, s, H-29), 0.88 (3H, s, H-31), 0.92 (3H, d, *J*=6.4 Hz, H-21), 0.98 (3H, s, H-19), 1.00 (3H, s, H-30), 1.02 (3H, d, *J*=6.9 Hz, H-26), 1.03 (3H, d, *J*=6.9 Hz, H-27), 2.23 (1H, dt, *J*=13.7, 6.8 Hz, H-25), 3.24 (1H, dd, *J*=11.7, 4.4 Hz, H-3), 4.66 (1H, br s, H-28a), 4.71 (1H, br s, H-28b);^{13}C NMR (100 MHz): Table 1. EIMS *m*/*z* (rel intensity): 440 $[M]^+$ (11), 425 (16), 422 (58), 407 (68), 393 (23), 379 (21), 353 (16), 325 (28), 297 (27), 295 (26), 281 (23), 255 (17); HREIMS: *m*/*z* 440.4000. $[M]^+$, calcd for $C_{31}H_{52}O$, 440.4018.

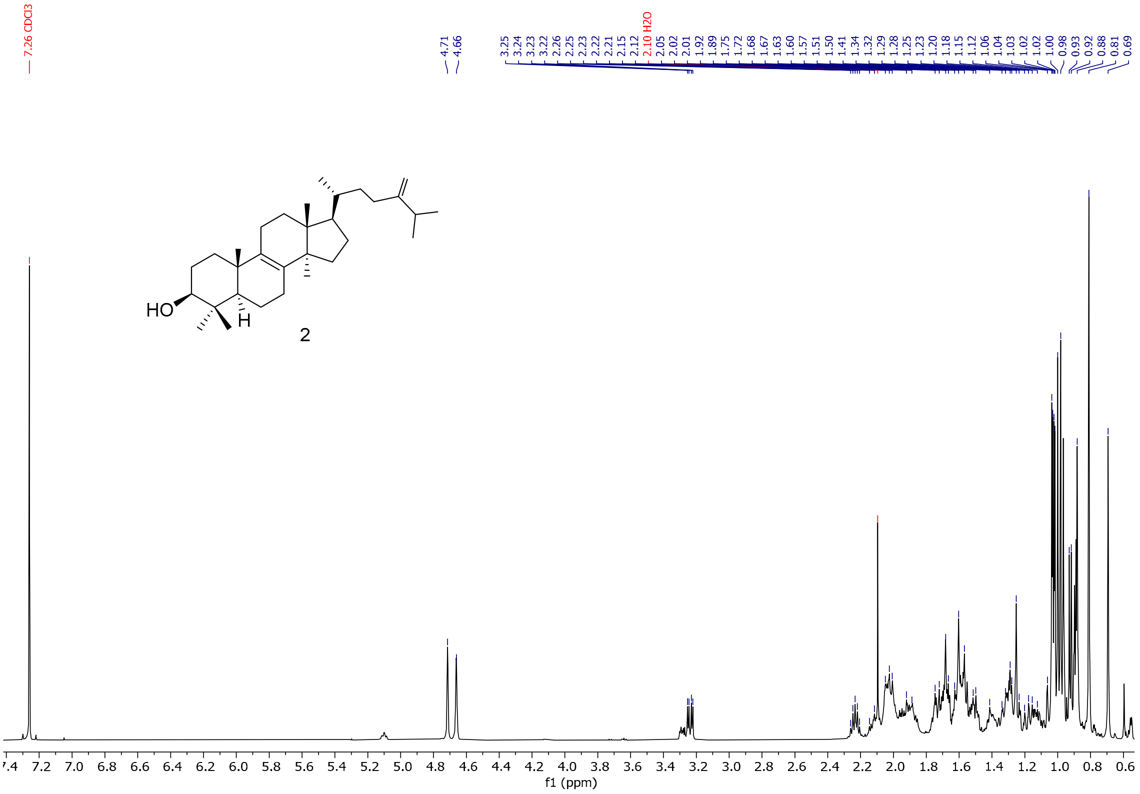

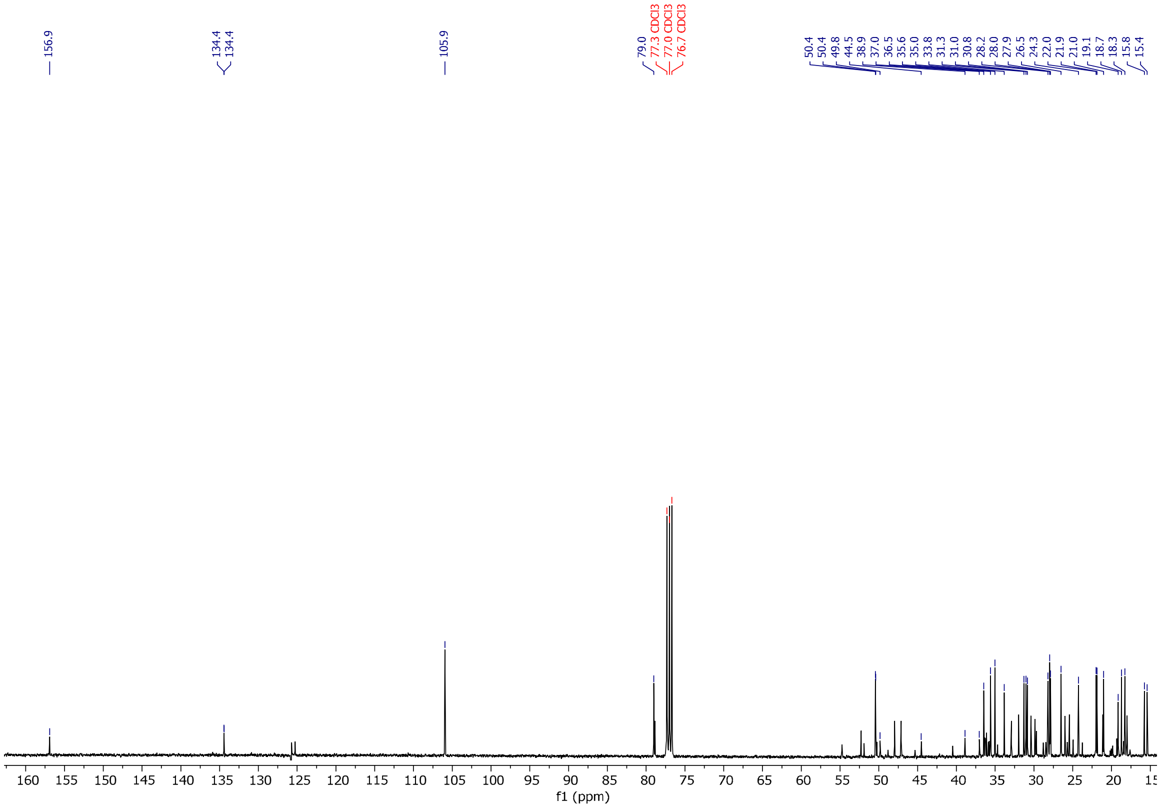

Figure S2. ^{1}H and ^{13}C NMR spectra of compound **2**

Elemental Composition Report Page 1

Multiple Mass Analysis: 866 mass(es) processed - displaying only valid results

[redacted]

Figure S3. HREIMS spectrum of compound **2**

Compound **3:**

^{1}H NMR (500 MHz, $CDCl_3$): δ 0.71 (3H, s, H-18), 0.81 (3H, d, *J*=6.8 Hz, H-26), 0.83 (3H, d, *J*=6.8 Hz, H-27), 0.85 (3H, t, *J*=7.4 Hz, H-29), 0.92 (3H, d, *J*=6.5 Hz, H-21), 1.18 (3H, s, H-18), 1.68 (1H, dd, *J* = 13.9, 4.8 Hz, H-25), 5.72 (1H, s, H-4); ^{13}C NMR (100 MHz): Table 1. EIMS *m*/*z* (rel intensity): 412 $[M]^{+}$ (51), 398 (25), 370 (17), 289 (20), 271 (18), 229 (39), 189 (12), 175 (18), 159 (13), 149 (36), 124 (100); HREIMS: *m*/*z* 412.3701 $[M]^{+}$, calcd for $C_{29}H_{48}O$, 412.3705.

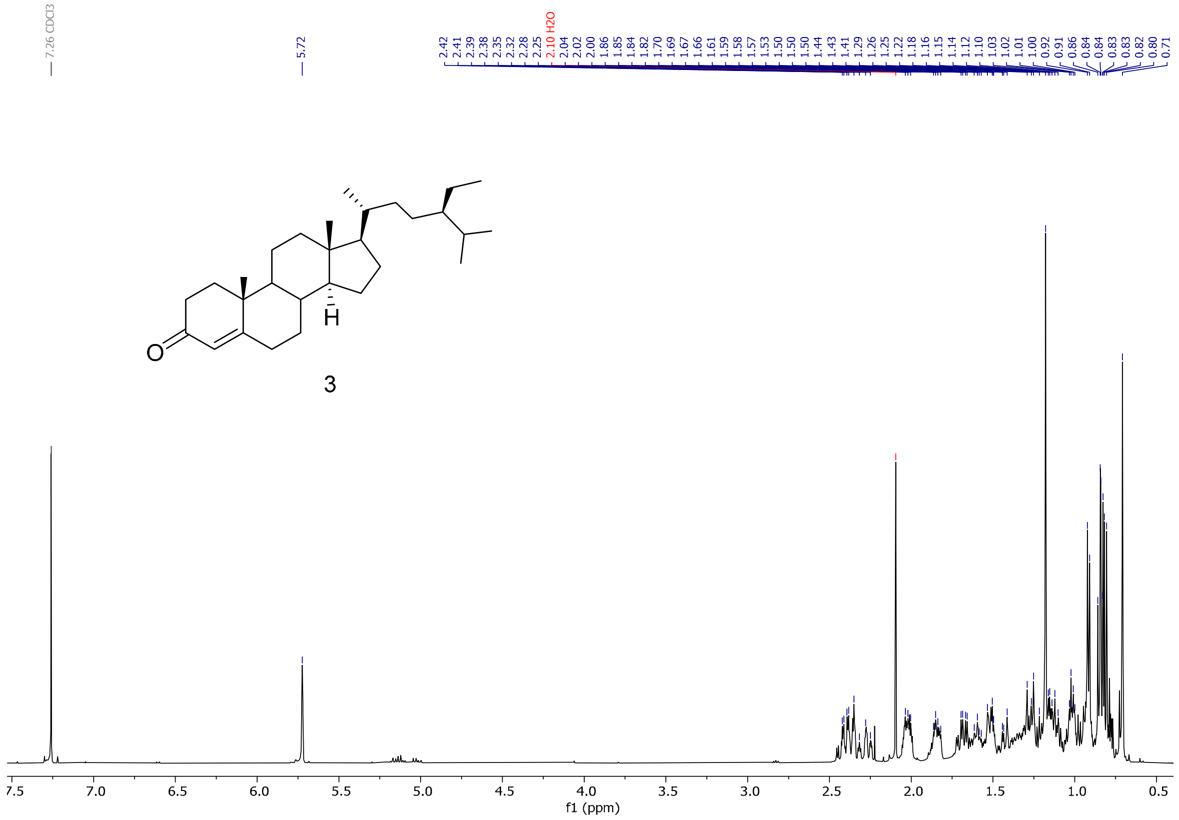

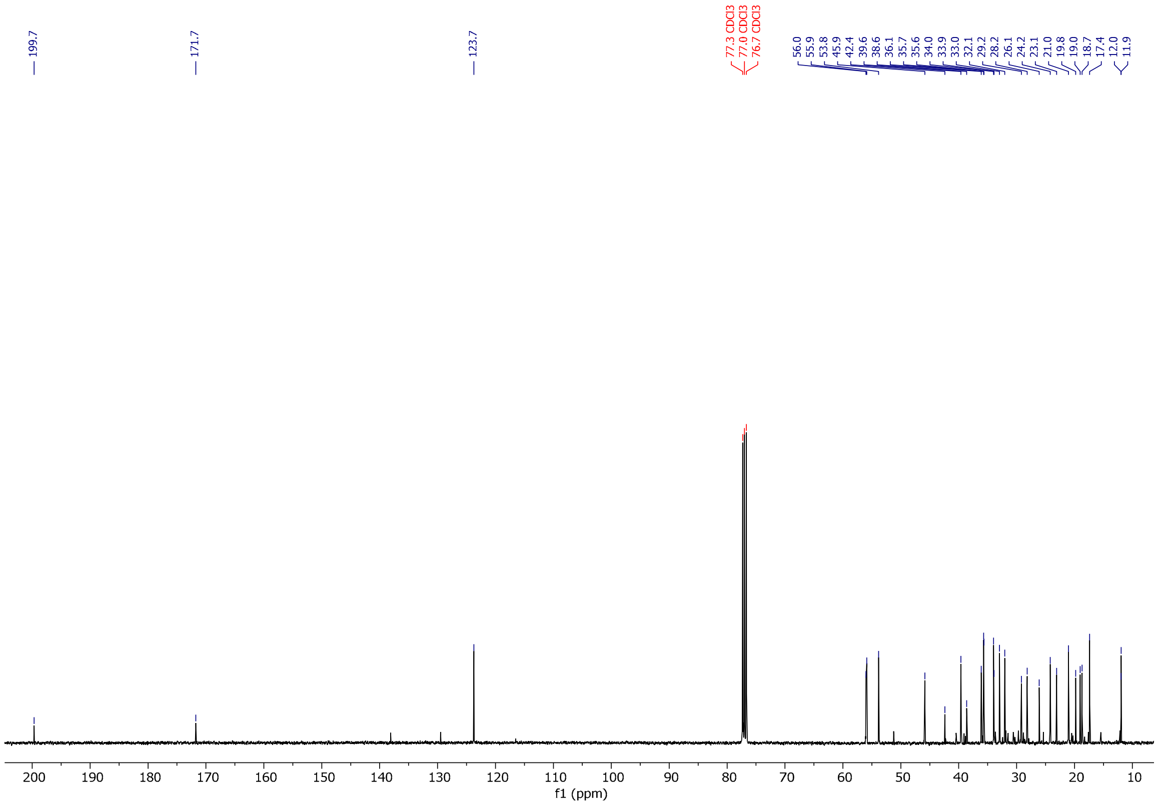

Figure S4. ^{1}H and ^{13}C NMR spectra of compound **3**

Elemental Composition Report **Page 1**

Multiple Mass Analysis: 69 mass(es) processed - displaying only valid results
Tolerance = 10.0 PPM / DBE: min = -1.5, max = 50.0
Selected filters: None

Monoisotopic Mass, Odd and Even Electron Ions
96 formula(e) evaluated with 3 results within limits (all results (up to 1000) for each mass)
Elements Used:
C: 0-29 H: 0-48 O: 0-1

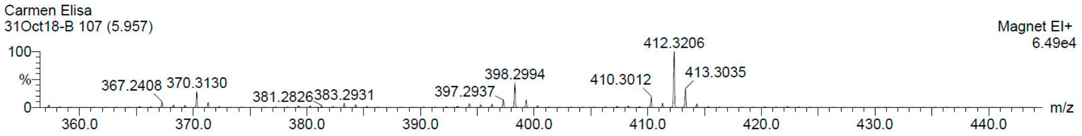

Minimum:	28.00				-1.5	
Maximum:	100.00		5.0	10.0	50.0	
Mass	RA	Calc. Mass	mDa	PPM	DBE	Formula
412.3701	33.35	412.3705	-0.4	-1.0	6.0	C29 H48 O
398.2627	29.11	398.2610	1.7	4.3	13.0	C29 H34 O
398.2590	28.53	398.2610	-2.0	-5.0	13.0	C29 H34 O

Figure S5. HREIMS spectrum of compound **3**

Compound **4:**

^{1}H NMR (500 MHz, $CDCl_3$): δ 0.63 (3H, s, H-18), 0.82 (3H, d, *J*=6.8 Hz, H-26), 0.84 (3H, d, *J*=6.8 Hz,H-27), 0.92 (3H, d, *J*=6.8 Hz, H-28), 0.94 (3H, s, H-19), 1.04 (3H, d, *J*=6.7 Hz, H-21), 2.28 (1H, t, *J*=12.9 Hz, H-4), 2.47 (1H, ddd, *J*=14.3, 4.8, 2.4 Hz, H-4), 3.64 (1H, tt, *J*=11.2, 4.3 Hz, H-3), 5.20 (2H, dd, *J*=11.4, 7.4 Hz, H-22, H-23), 5.38 (1H, dt, *J*=5.6, 2.8 Hz, H-7), 5.57 (1H, dd, *J*=5.7, 2.6 Hz, H-6);^{13}C NMR (100 MHz, $CDCl_3$): Table 1. EIMS *m*/*z* (rel intensity): 396 $[M]^+$ (100), 363 (58), 337 (23), 253 (18), 211 (12), 171 (9), 159 (12), 157 (13), 143 (9), 109 (6), 83 (9), 81 (13); HREIMS: *m*/*z* 396.3381 $[M]^+$, calcd for $C_{28}H_{44}O$, 396.3392.

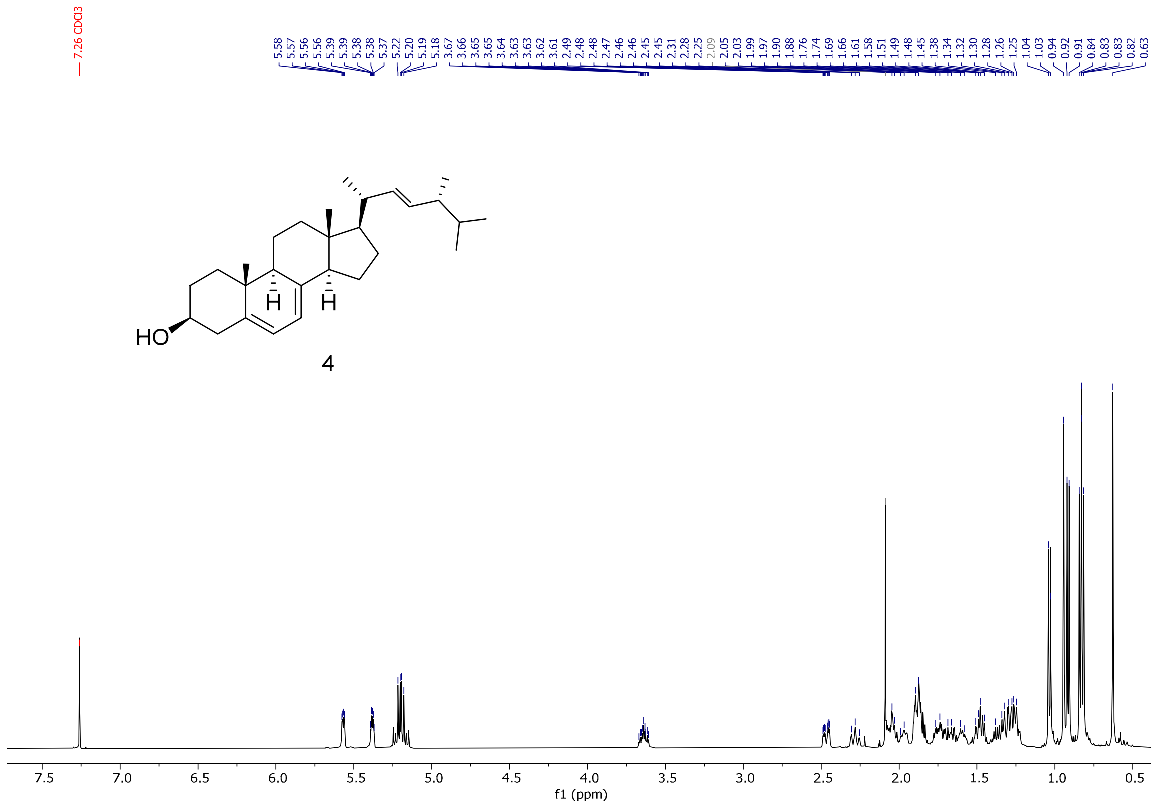

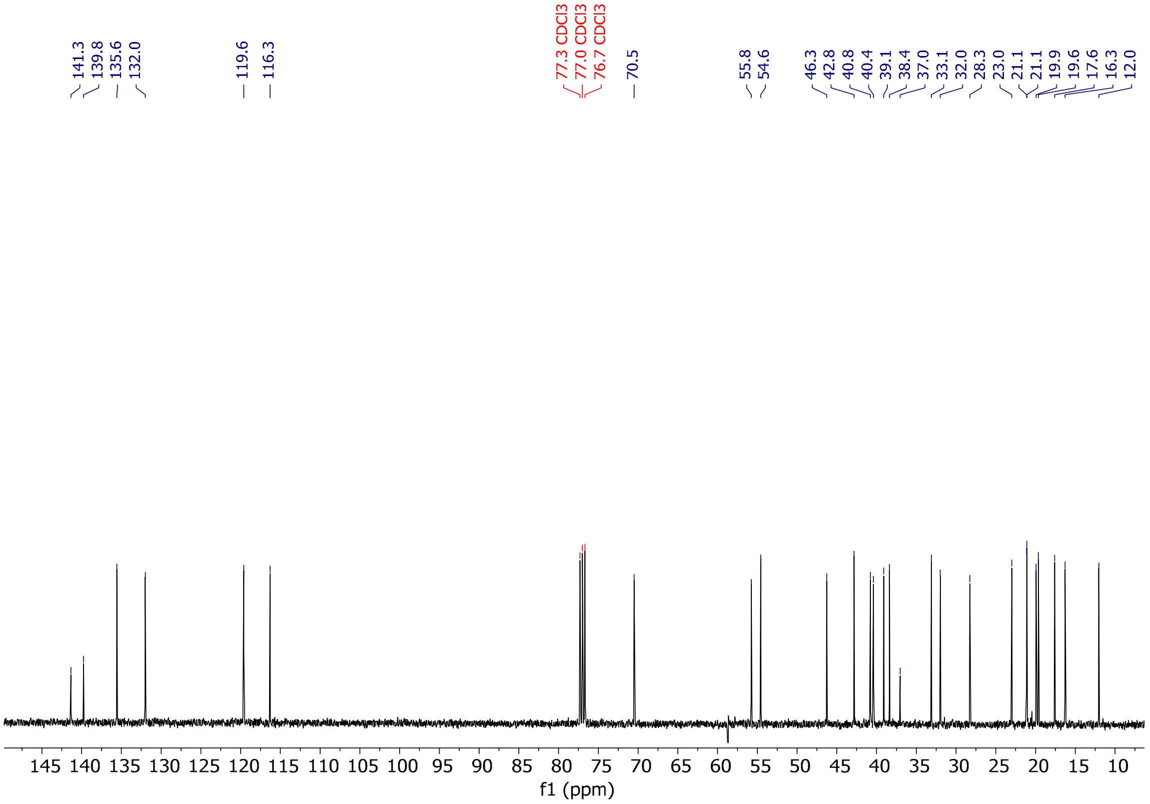

Figure S6. ^{1}H and ^{13}C NMR spectra of compound **4**

Elemental Composition Report **Page 1**

Multiple Mass Analysis: 246 mass(es) processed - displaying only valid results

Tolerance = 10.0 PPM / DBE: min = -1.5, max = 50.0

Selected filters: None

Monoisotopic Mass, Odd and Even Electron Ions

382 formula(e) evaluated with 1 results within limits (all results (up to 1000) for each mass)

Elements Used:

C: 28-28 H: 10-44 O: 0-1

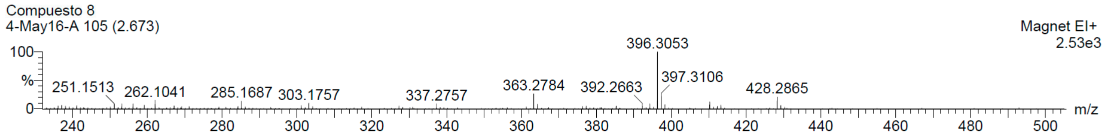

Minimum:	4.50				-1.5	
Maximum:	100.00		5.0	10.0	50.0	
Mass	RA	Calc. Mass	mDa	PPM	DBE	Formula
396.3381	5.65	396.3392	-1.1	-2.8	7.0	C28 H44 O

Figure S7. HREIMS spectrum of compound **4**

Compound **5:**

^{1}H NMR (500 MHz, $CDCl_3$): δ 0.81 (3H, s, H-18), 0.82 (3H, d, *J*=6.8 Hz, H-26), 0.83 (3H, d, *J*=6.8 Hz, H-27), 0.88 (3H, s, H-19), 0.91 (3H, d, *J*=6.8 Hz, H-28), 1.00 (3H, d, *J*=6.6 Hz, H-21), 3.97 (1H, ddd, *J*=16.5, 11.6, 5.1 Hz, H-3), 5.14 (1H, dd, *J*=15.3, 8.3 Hz, H-23), 5.22 (1H, dd, *J*=15.3, 7.6 Hz, H-22), 6.24 (1H, d, *J*=8.5 Hz, H-6), 6.50 (1H, d, *J*=8.5 Hz, H-7);^{13}C NMR (125 MHz, $CDCl_3$): Table 1. EIMS *m*/*z* (rel intensity): 428 $[M]^+$ (7), 396 (42), 363 (9), 337 (4), 285 (4), 262 (7), 218 (6), 203 (6), 175 (19), 159 (14), 152 (16), 129 (20), 109 (28), 97 (27), 95 (30), 83 (42), 81 (44); HREIMS: *m*/*z* 428.3274 $[M]^+$, calcd for $C_{28}H_{44}O_3$, 428.3290.

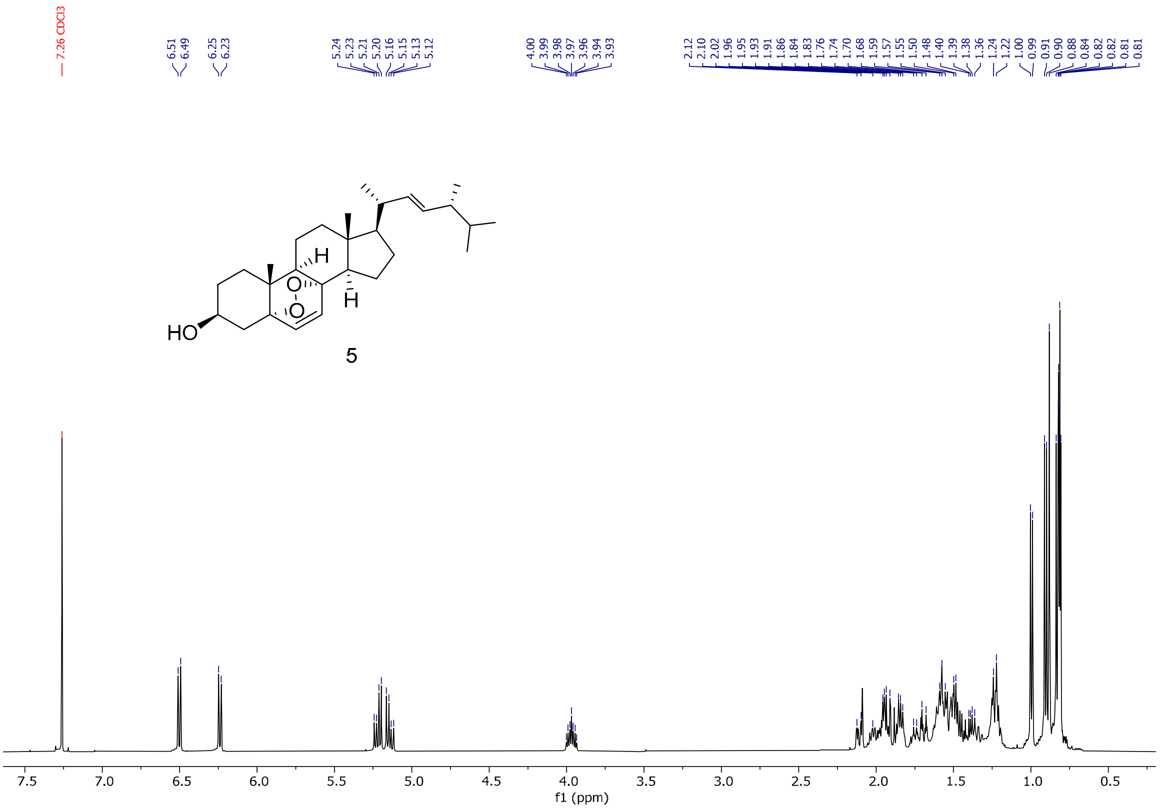

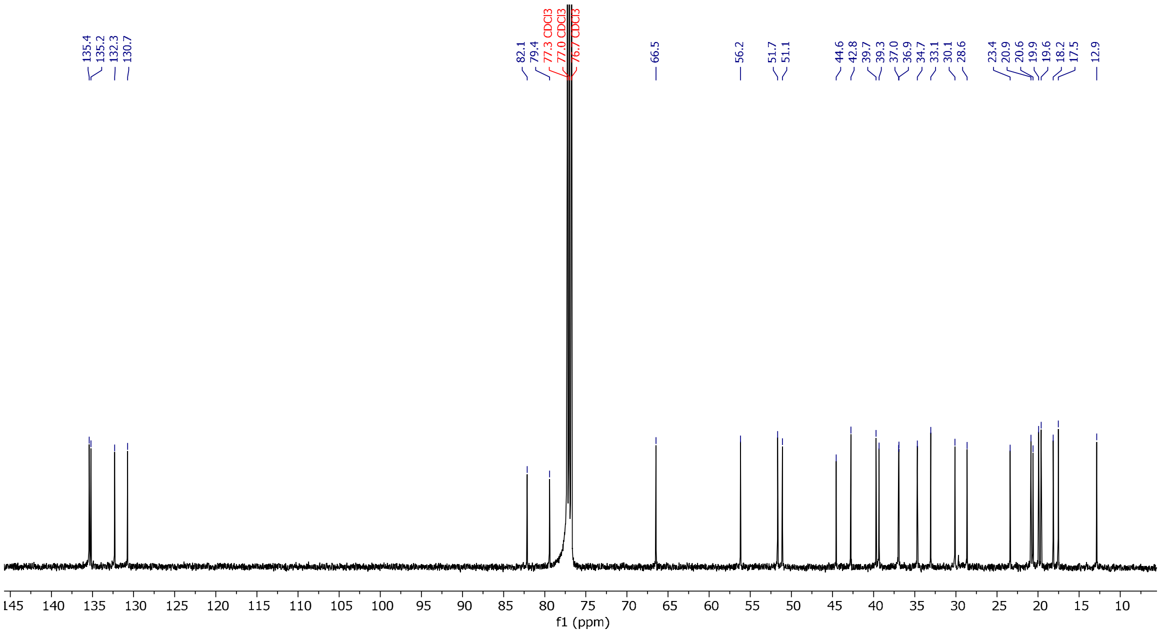

Figure S8. ^{1}H and ^{13}C NMR spectra of compound **5**

Elemental Composition Report **Page 1**

Multiple Mass Analysis: 102 mass(es) processed - displaying only valid results
Tolerance = 10.0 PPM / DBE: min = -1.5, max = 50.0
Selected filters: None

Monoisotopic Mass, Odd and Even Electron Ions
78 formula(e) evaluated with 1 results within limits (all results (up to 1000) for each mass)
Elements Used:
C: 28-28 H: 10-44 O: 3-3
Compuesto 8
4-May16-AAFAMM 102 (2.596) Magnet EI+ 1.66e3

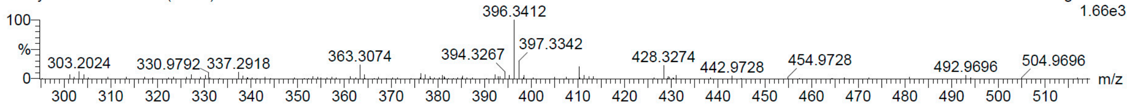

Minimum:	0.60				-1.5		
Maximum:	100.00		5.0	10.0	50.0		
Mass	RA	Calc. Mass	mDa	PPM	DBE	i-FIT	Formula
428.3274	22.54	428.3290	-1.6	-3.7	7.0	16.1	C28 H44 O3

Figure S9. HREIMS spectrum of compound **5**

Carbon	**2**	**3**	**4**	**5**
1	35.6	35.6	38.4	34.7
2	27.9	33.9	32.0	30.1
3	79.0	199.7	70.5	66.5
4	38.9	123.7	40.8	37
5	50.4	171.7	139.8	79.4
6	18.3	33.0	119.6	135.4
7	26.5	32.1	116.3	130.7
8	134.4	35.7	141.3	82.1
9	134.4	53.8	46.3	51.1
10	37.0	38.6	37.0	36.9
11	21.0	21.0	21.1	20.6
12	31.0	39.6	39.1	39.3
13	44.5	42.4	42.8	44.6
14	49.8	55.9	54.6	51.7
15	30.8	24.2	23.0	23.4
16	28.2	28.2	28.3	28.6
17	50.4	56.0	55.7	56.2
18	15.8	11.9	12.0	12.9
19	19.1	17.4	16.3	18.2
20	36.5	36.1	40.4	39.7
21	18.7	18.7	21.1	20.9
22	35.0	34.0	135.6	132.5
23	31.3	26.1	132.0	135.2
24	156.9	45.9	42.8	42.8
25	33.8	29.2	33.1	33.1
26	22.0*	19.8*	19.8*	19.9*
27	21.0*	19.0*	19.6*	19.6*
28	105.9	23.1	17.6	17.5
29	15.4	12.0		
30	28.0			
31	24.3			

*data interchangeable

Table S1. ^{13}C NMR data of compounds **2-5**

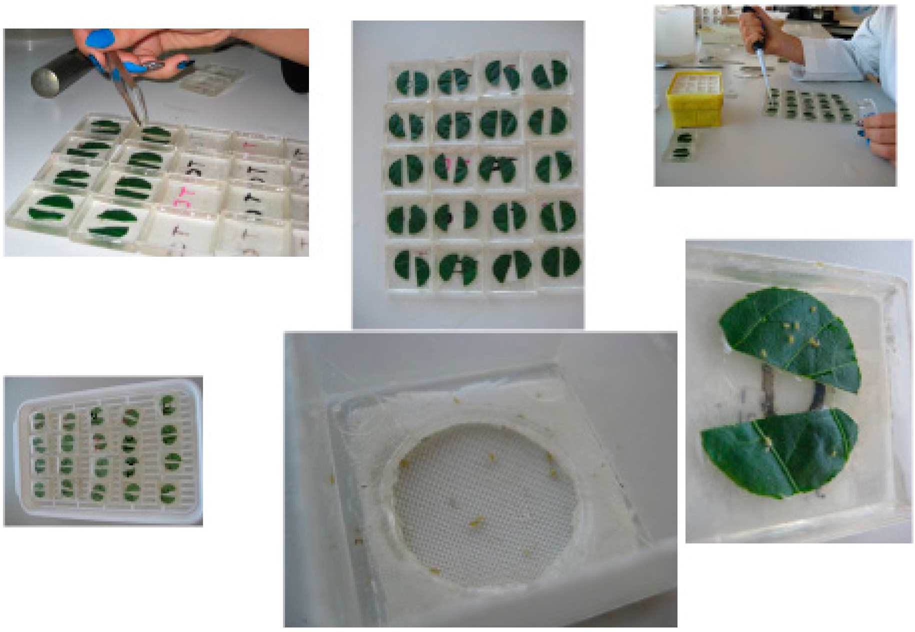

Figure S10. Antifeedant bioassay against *Myzus persicae*

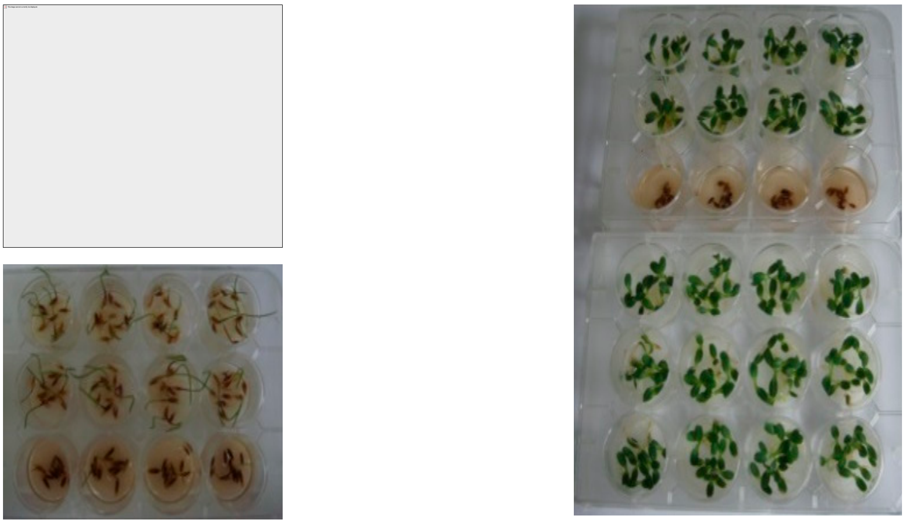

Figure S11. Phytotoxicity bioassay against *Lolium perenne* and *Lactuca sativa*
